# Balance Impairments in Patients with Human T-Cell Lymphotropic Virus Type 1 Infection

**DOI:** 10.1038/s41598-019-47920-z

**Published:** 2019-08-07

**Authors:** Beatriz Helena B. Vasconcelos, Bianca Callegari, Kelly Helorany A. Costa, Tatiana G. C. P. Barroso, Rita Catarina M. Sousa, Ghislain Saunier, Marília B. Xavier, Givago S. Souza

**Affiliations:** 1 Laboratory of Human Motricity, Federal University of Pará, Belém, Pará Brazil; 2 Institute of Biological Sciences, Federal University of Pará, Belém, Pará Brazil; 3 Tropical Medicine Nucleus, Federal University of Pará, Belém, Pará Brazil; 4 Laboratory of Motor Cognition, Federal University of Pará, Belém, Pará Brazil

**Keywords:** Viral infection, Viral infection, Central nervous system infections, Central nervous system infections, Motor neuron disease

## Abstract

The human T-cell lymphotropic virus type 1 (HTLV-1) is a retrovirus from the *Retroviridae* family that infects cluster of differentiation 4 (CD4) T-lymphocytes and stimulates their proliferation. A severe consequence of this infection can be the HTLV-1 associated myelopathy/tropical spastic paraparesis (HAM/TSP), which is associated with a progressive demyelinating disease of the upper motor neurons. The HAM/TSP conditions frequently present with neurological complaints such as gait impairment, sphincter disturbances, and several sensory losses. We compared findings from the posturographic evaluation from the asymptomatic HTLV-1 infected subjects, HTLV-1 infected subjects having HAM/TSP, and control group database. A force plate was used to record the postural oscillations. Analysis of variance and multivariate linear discriminant analysis were used to compare the data obtained from the three groups of participants. In general, HAM/TSP patients had worse postural balance control than did the HTLV-1 patients and the controls (p < 0.05). We found that in six out of ten parameters of the postural balance control, there was a gradual increase in impairment from control to HTLV-1 to HAM/TSP groups. All parameters had higher values with the subject’s eyes closed. The multivariate linear discriminant analysis showed there was a reasonable difference in results between the control and HAM/TSP groups, and the HTLV-1 group was at the intersecting area between them. We found that HAM/TSP patients had worse balance control than did HTLV-1 infected patients and the control group, but asymptomatic HTLV-1 infected patients represent an intermediate balance control status between controls and HAM/TSP patients. Posturographic parameters can be relied on to identify subtle changes in the balance of HTLV-1 patients and to monitor their functional loss. HTLV-1 is a tropical disease that can be transmitted by sexual intercourse, blood transfusion, and breast-feeding. Some infected subjects develop an HTLV-1-associated myelopathy/tropical spastic paraparesis (HAM/TSP), a condition characterized by spasticity, weakness in lower limbs, and difficulty in walking long distances and going up and down the stairs, besides the history of falls. We compared the body oscillations using a force plate to investigate the postural balance control. HTLV-1 infected patients had imbalance that could be identified by posturographic parameters. Patients with HAM/TSP clearly had balance impairments, while HTLV-1 without HAM/TSP had a subtle impairment that was not seen on clinical scales, suggesting that these patients were in the middle between healthy and HAM/TSP patients, and carried a risk of developing severe imbalance postural control. We suggest that more research should be done with the aim to identify the subtle signs in asymptomatic HTLV-1 patients to investigate if this group of patients need attention similar to the HAM/TSP patients.

## Introduction

The human T-cell lymphotropic virus type 1 (HTLV-1) is a retrovirus from the *Retroviridae* family that infects cluster of differentiation 4 (CD4) T-lymphocytes and stimulates their proliferation^1^. The most severe consequences of HTLV-1 infection are adult T-cell leukemia/lymphoma (ATL) and HTLV-1 associated myelopathy/tropical spastic paraparesis (HAM/TSP)^1–4^. HAM/TSP is a progressive demyelinating disease of the upper motor neurons^5–13^, and it affects 0.3 to 4% of HTLV-1 infected individuals, depending on the geographic location^14^.

Since HAM/TSP progression includes the degeneration of motor neurons, progressive spastic paraparesis, or paraplegia is the main clinical finding for HAM/TSP leading to significant gait impairment and functional disability^14^. Low back pain, sphincter disturbances, and other several sensory losses may also be observed^10,15–17^. In a previous study, we demonstrated that the prevalence of neurological disorders (i.e. altered reflexes, skin tactile sensitivity, and increased risk of falling) was higher in HTLV-1 HAM/TSP patients than in HTLV-1 asymptomatic patients^18^. Besides, the asymptomatic HTLV-1 infection caused a displacement of the body weight from the hindfoot to the forefoot, and it was remarkably higher in HAM/TSP patients. The pressure values of HTLV-1 patients were intermediate, between the values measured in controls and HAM/TSP patients, suggesting a gradual increase in impairment of these HTLV-1 infection-related complications towards the more severe phenotype observed in HAM/TSP patients^18^.

This abnormal pattern of body weight distribution on the forefoot is closely related to balance control^19,20^. The ability to maintain balance is an essential requirement for activities of daily living (ADL)^21^.

Balance impairments in HAM/TSP patients are rarely reported^22,23^. These previous investigations have major limitations since the balance assessments were performed only using clinical scales, like BERG and TINETTI. These tools, besides largely used, commonly fail to detect differences in the balance at higher values of the range^24,25^. Other more objective evaluation techniques apply a quantitative analysis of the center of pressure (COP) using posturography, which measures the forces exerted against the ground from a force platform during a static position^21^. These parameters are commonly used to quantify postural steadiness both for research and clinical purposes. Ordinarily, posturography focuses on the properties of the time-series changes of the COP, representing the point of location of the ground reaction force vector as it evolves in the horizontal plane or along two orthogonal axes, fixed with the platform in anteroposterior and mediolateral deviations across the time^26^.

The present investigation aimed to quantify balance control in HTLV-1 patients, with and without HAM/TSP, using a force platform and comparing them to healthy subjects. Our hypothesis was that the HAM/TSP patients (with sensorial and motor impairments) would be more prone to postural imbalance than the asymptomatic HTLV-1 patients or the control subjects.

## Methods

### Subjects

For the current investigation, the sample size was fifty-three participants for this study. The participants were divided into three groups: HTLV-1 group was composed of 18 patients; HAM/TSP group was composed of 18 patients; and control group was composed of 17 healthy subjects (characteristics presented in Table 1). All participants gave written consent for both study participation and publication of identifying information/images. Ethics Committee for Research with Human Subjects, Institute of Health Sciences, Federal University of Pará approved the procedures carried out in the present investigation (report #633.187). All methods were performed in accordance with the Declaration of Helsinki.

**Table 1:** Characteristics of the subjects in the three experimental groups.

	Control	HTLV-1	HAM/TSP
Sex	13F, 4M	14F, 4M	10F, 8M
Age (years)	44.8 ± 10.5	46 ± 11.7	51 ± 9.7
Weight (kg)	68.5 ± 18.2	65 ± 11.2	62.7 ± 8.1
Height (m)	1.57 ± 0.1	1.55 ± 0.08	1.57 ± 0.07
BMI	27.2 ± 5.4	26.8 ± 3.8	25.3 ± 3.3
Time since diagnoses (months)	—	74.2 ± 74.8	67.4 ± 48.3*

*p < 0.05. F, female. M, male. BMI, body mass index. Data are presented as mean ± standard deviation (SD).

The HTLV-1 infected subjects were diagnosed in the Tropical Medicine Nucleus of the Federal University of Pará, Brazil. For the diagnosis, we considered the World Health Organization criteria: an infectologist collected data on the patient’s clinical history, neurological evaluation results, and results of seropositivity using the ELISA method (Cambridge Biotech, Worcester, MA, USA), Western blot analysis (HTLV blot 2.4, Genelab, Singapore), polymerase chain reaction (PCR), or a combination of these examinations for HTLV-1 infection.

We excluded from the sample patients unable to remain in standing position without assistance, those having neurological disorders with diagnoses other than HTLV-1 infection, such as diabetes, rheumatic diseases, and peripheral vestibular syndrome; HIV-coinfected patients; and pregnant women.

### Clinical evaluation

We evaluated the patellar and Achilles reflexes using a reflex hammer. The skin tactile sensitivity was measured using 5 monofilaments with force levels of 0.2, 2, 4, 10, and 300 g in 8 different areas of the foot (6 areas in the forefoot, 1 in the midfoot, and 1 in the hindfoot). The test was carried out for three trials starting from the lightest to the heaviest monofilament. The lightest monofilament identified by the subject was considered the threshold. Thresholds higher than 0.2 g were considered as an altered result. We used the BERG balance scale to evaluate the dynamic balance, which includes tasks of postural transfers, stationary balance during sitting and standing positions, functional reach, rotational components, and base of support. The maximum score for the scale is 56, and the risk of falling was classified as low (scores > 41), medium (scores from 21 to 40), or high (scores < 20).

### Static balance performance

We used a force platform (BIOMEC400, EMG System do Brasil, Ltda., SP, Brazil), with charged sensors distributed at 50 cm², connected to a computer with Biomec software (EMG System do Brasil, Ltda., SP, Brazil). Environmental illumination and sound conditions were kept constant during the evaluation of all subjects. The static balance analyses were performed with the individuals standing barefoot, with feet shoulder-width apart, and arms resting along the body. The test was performed for 1 minute each with the eyes closed (3 trials) and eyes opened (3 trials). With the eyes opened, the subjects gazed a target with black cross target painted on the wall 1 m away. A period of 30 to 60 seconds of rest separated two consecutive recording sessions. The displacements of the COP in the anterior-posterior (AP) and in a medio-lateral (ML) planes as a function of the time were recorded.

### Data analysis

From the COP displacement time-series, we used the Biomec software (EMG System do Brasil, Ltda., SP, Brazil) to extract the total COP displacement, AP and ML amplitudes, total area of the COP displacement, AP and ML velocities, AP and ML median frequencies, and AP and ML mean frequencies. The total COP displacement was the resultant vector of the displacements in the AP and ML planes. AP and ML amplitudes were the highest amplitude displacements in the AP and ML planes, respectively. The total area of the COP displacement was calculated by fitting an ellipse to the COP displacements covering 90% of all displacement data points. The AP and ML velocities were calculated as the COP displacement in the AP and ML planes divided by the time unit, respectively. The AP and ML median frequencies were the temporal frequencies that divided the Fourier power spectrum domain related to the AP and ML displacement in two equal parts, respectively. The AP and ML mean frequencies were calculated using the Equation (1).

$$Mean\,frequency=\frac{{\sum }_{i}^{m}\,{f}_{i}\times {P}_{i}}{{\sum }_{i}^{m}\,{P}_{i}}$$

where *f* is the *ith* temporal frequency of the Fourier power spectrum domain, *P* is the power of the *ith* temporal frequency, *m* is the number of frequency bins.

We averaged the three trials of each test condition and we used it for statistical analyses. One-way ANOVA followed by post-hoc test was used to evaluate the effect of the factor group (HTLV-1, HAM/TSP, and control groups) on the COP displacement parameters in open and closed eye conditions. We also carried out a multivariate linear discriminant analysis (LDA) to extract the features that maximize the difference in distance between-groups and minimize the distance within-groups. All the results of the posturographic parameters were used as inputs in the multivariate analysis. All statistical analyses were performed using R-Plus (RStudio, Vienna, AUS) with the RStudio Interface (RStudio Team, Boston, MA, USA).

## Results

### Clinical evaluation

The control group was matched to the HTLV-1-infected patients in age, sex, and body mass index (BMI) (for age comparison: F[2, 51] = 1.44, p = 0.24); for BMI comparison (F[2, 51] = 1.48, p = 0.23). differences among all the groups. We found 5 HTLV-1 and 9 HAM/TSP patients with altered patellar and Achilles reflexes, and other 2 HAM/TSP patients with single altered patellar reflex. We observed HTLV-1 patients with reduced tactile sensitivity in the forefoot (12 patients) and in the hindfoot (14 patients). HAM/TSP patients also had loss of tactile sensitivity in the forefoot (17 patients) and hindfoot (16 patients). All the HTLV-1 patients had BERG scores associated with a low risk of falling, and HAM/TSP patients had BERG scores associated with low (nine patients), medium (eight patients), and high (one patient) risk of falling.

### Posturography

**Figure 1:**
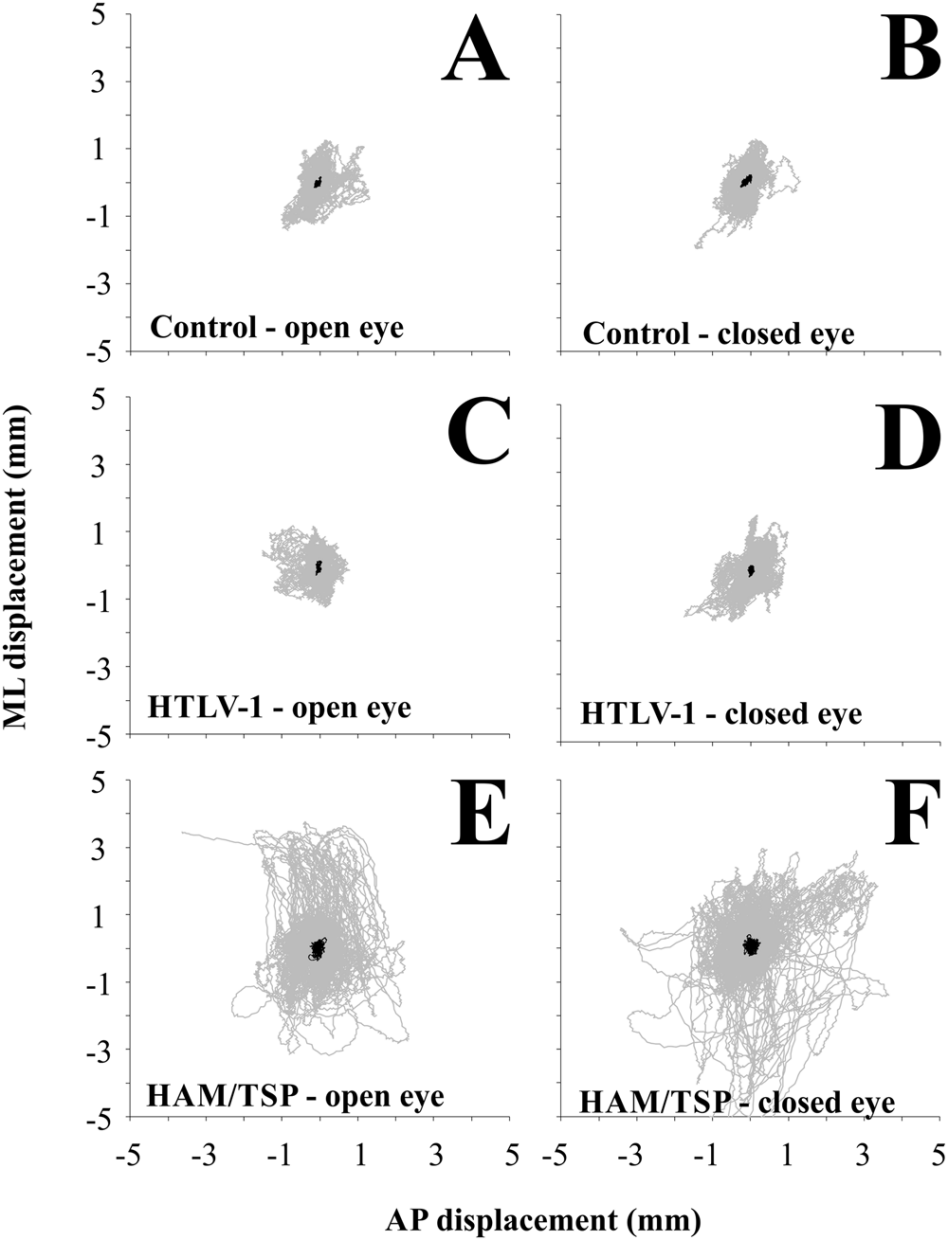
Overlapped statokynesiograms for control, HTVL-1, and HAM/TSP groups in open eye condition (**A**,**C**,**E** respectively), and closed eye condition (**B**,**D**,**F** respectively). Gray lines represent the individual recordings, black lines represent the averaged data.

Figure 1 compares the postural balance in AP and ML planes and the statokynesiogram of the control group and HTLV-1 patients (asymptomatic and symptomatic patients) with eyes open and eyes closed. We observed that PET-MAH patients had more oscillations than did the other two groups.

Figure 1A,C,E show the overlapped COP displacements time series in the AP (blue lines) and ML (red lines) with eyes open for all the control, HTLV-1 group, and HAM/TSP group participants. We could observe a gradual increase in the displacements from the control group to the HAM/TSP group. The statokinesiograms showed in the Fig. 1B,D,F also allow to do the same observation when experiment was carried out with eyes closed. The results suggested similar interpretation as Fig. 1.

Table 2 compares the mean values for posturography among the three groups of the present study. In general, the HAM/TSP group had worse results than the HTLV-1 group and the control group. Comparison showed no difference with closed and open eyes between HTLV-1 group and control group, but for at least three parameters: AP and ML amplitudes and area, nevertheless the absence of statistical significance, we observed that the HTLV-1 group results were in the mid-range between the controls and HAM/TSP group.

**Table 2:** Parameters of posturography estimated with eyes open (OE) and eyes closed (CE).

Posturographic parameters		Control	HTLV-1	HAM/TSP
		Mean ± SD	Mean ± SD	Mean ± SD
AP amplitude (cm)	OE	1.21 ± 0.4	1.26 ± 0.4	1.78 ± 0.7^a^
	CE	1.25 ± 0.3	1.32 ± 0.43	2.18 ± 0.98^a^
ML amplitude (cm)	OE	0.58 ± 0.17	0.64 ± 0.24	1.12 ± 0.48^a^
	CE	0.66 ± 0.23	0.84 ± 0.43^b^	1.21 ± 0.48^a^
Total displacement (cm)	OE	32.91 ± 3.7	33.31 ± 7.1	46 ± 15.6^a^
	CE	39.44 ± 5.2	39.13 ± 10.1^a^	61.41 ± 29.8^c^
Area (cm²)	OE	0.4 ± 0.18	0.63 ± 0.6	1.63 ± 1.4^a^
	CE	0.47 ± 0.2	0.69 ± 0.6	1.81 ± 1.6^a^
AP velocity (cm/s)	OE	0.48 ± 0.04	0.39 ± 0.1	0.63 ± 0.3^a^
	CE	0.49 ± 0.1	0.41 ± 0.1	0.73 ± 0.3^a^
ML velocity (cm/s)	OE	0.33 ± 0.05	0.34 ± 0.1	0.41 ± 0.1^a^
	CE	0.35 ± 0.04	0.37 ± 0.1	0.46 ± 0.1^a^
AP median frequency (Hz)	OE	0.14 ± 0.1	0.16 ± 0.1	0.24 ± 0.2
	CE	0.22 ± 0.1	0.23 ± 0.1	0.30 ± 0.1
ML median frequency (Hz)	OE	0.24 ± 0.1	0.23 ± 0.1	0.24 ± 0.1
	CE	0.31 ± 0.1	0.24 ± 0.1	0.28 ± 0.1
AP mean frequency (Hz)	OE	0.24 ± 0.1	0.26 ± 0.1	0.34 ± 0.2
	CE	0.36 ± 0.1	0.33 ± 0.1	0.38 ± 0.1
ML mean frequency (Hz)	OE	0.46 ± 0.2	0.39 ± 0.2	0.34 ± 0.1
	CE	0.5 ± 0.2	0.4 ± 0.2	0.37 ± 0.1

^a^p < 0.05 HAM/TSP differed from HTLV-1 and control groups; ^b^p < 0.05 HAM/TSP differed from HTLV-1 group; ^c^p < 0.05 HAM/TSP differed control group. EO: eyes opened; EC: eyes closed; ML: medio-lateral; AP: antero-posterior; SD: standard deviation.

**Figure 2:**
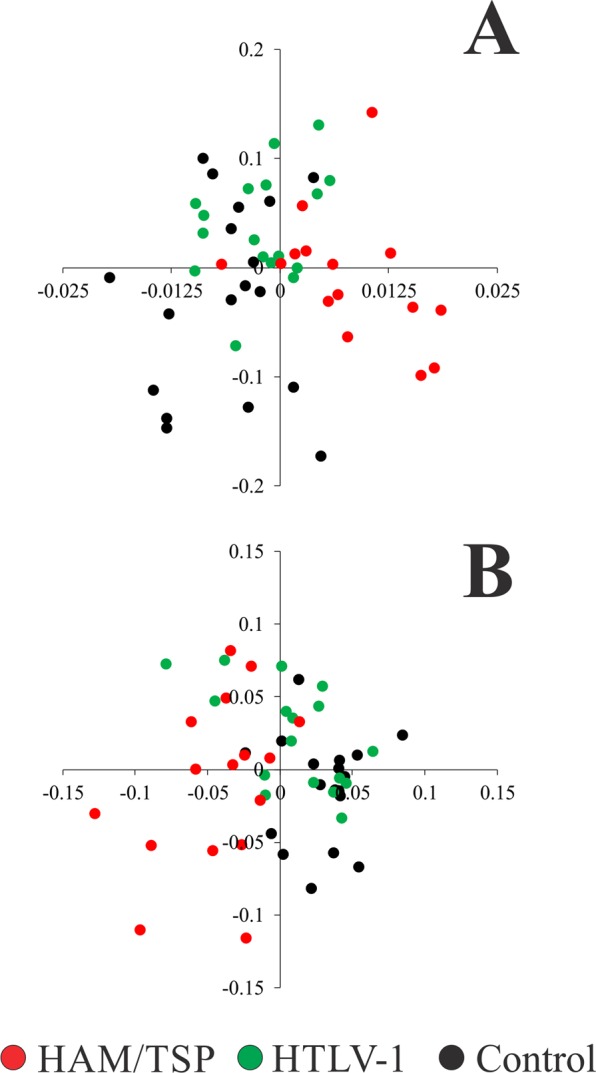
Multivariate linear discriminant analysis results for open eye and closed eye conditions (**A**,**B**, respectively). Controls (black circles) were in the opposite side of the HAM/TSP group (red circles) data in the bidimensional. HTLV-1 data (green circles) were found in a transition between controls and HAM/TSP database.

Multivariate linear discriminant analysis was applied for the results with eyes open and eyes closed. Table 3 shows the canonical coefficients for LDA in both test conditions. Velocity parameters had highest coefficients of the multivariate analysis with eyes open, while the frequency parameters had the highest coefficients of the multivariate analysis for eyes closed. We observed that for both test conditions (Fig. 2), there was separation between the control and HAM/TSP groups, and the HTLV-1 group was found at their intersections.

**Table 3:** Canonical coefficients estimated from multivariate linear discriminant analysis.

Variables	Canonical coefficients			
	With Eyes Open		With Eyes Closed	
	F1	F2	F1	F2
AP amplitude	0.0049	−0.1524	0.0407	0.0133
ML amplitude	0.0025	−0.3531	0.014	−0.0877
Total displacement	−0.0154	−0.003	−0.0033	−0.0044
Area	0.0064	0.2515	−0.0161	0.0063
AP velocity	0.8112	−0.3819	−0.002	−0.0015
ML velocity	0.5829	0.322	0.1168	0.5766
AP median frequency	−0.0025	0.5607	−0.6771	0.6569
ML median frequency	−0.0009	0.0298	−0.1806	−0.3739
AP mean frequency	0.0428	0.1723	0.6817	−0.2939
ML mean frequency	−0.0008	−0.4409	0.1687	−0.0434

F1 and F2 represent the first two discriminant functions from LDA.

## Discussion

The main objective of this study was to assess the balance control in HTLV-1 infected-patients with and without HAM/TSP, using a force platform. The present findings confirmed the clinical evidence that HAM/TSP patients have a condition of imbalance compared to controls. Additionally, we also observed that HTLV-1 patients reflect a transition status between control subjects and HAM/TSP patients.

We understand that some reports have described loss of the balance control in HTLV-1 infected patients^22–25^. Most of these studies used scales to quantify the balance control. The present manuscript is the first to describe a detailed quantitative description based in force platform measurements of the balance control loss in HTLV-1 patients.

Previously, we showed that the HAM/TSP patients exert greater pressure in the forefoot when compared to hindfoot, and the HTLV-1 patients without HAM/TSP were intermediate between the pressure values of HAM/TSP and control group^18^. The combined plantar pressure and stabilometric results indicate that HAM/TSP patients had a significant postural control deficit, which can lead to an increase in risk of falls; that asymptomatic HTLV-1 infected patients are sub-clinically susceptible to these balance impairments; and that they need continuous evaluations of their postural control. Other studies have found functional loss in HTLV-1 patients compared to the control group, such as altered plantar foot pressure^18^, hearing loss^27^, and impaired vestibular evoked myogenic potentials^28^. Nagai *et al*.^29^ suggested that high proviral load in HTLV-1 carriers could predispose them to develop symptoms of HAM/TSP. The increase of the proviral load may be related with the onset of an inflammatory process in the spinal cord, and as its consequence, functional complications can arise.

Among the many stabilometric parameters, we found the proportional change between controls and infected patients was higher in the ML amplitude parameter. Winter *et al*.^30^ reported that in static posture with feet parallel to the ground, the muscle groups of the hip are solely responsible for anticipatory adjustments to maintain the balance in the medio-lateral plane. Caiafa *et al*.^31^ identified the hip adductors and plantar flexors as the most affected muscles by weakness and spasticity in HTLV-1-associated myelopathy. The spasticity in hip adductors could elicit the increased oscillation in the medio-lateral plane as that we obtained in the present study. As we did no evaluation of spasticity in our patients, it is not possible to assert the relationship between the loss of ML balance control and hip muscle involvement, but our findings indicate a careful evaluation of this muscle group in HTLV-1 patients^31^.

The assessment using measures of COP recorded from a force platform are considered the gold standard for measuring balance^32^ because it can identify important outcome measures, which are too subtle to detect using a subject scale^33^. In this way, the force platform provides posturography parameters, such as the area of the COP displacement and ML amplitude, that can be used to show the profile of HTLV-1 patients, which can be used to differentiate between symptomatic and asymptomatic subjects. We used 10 different stabilometric parameters to feed a linear discriminant analysis and to extract the features that better determine the between-groups separation. However, when we applied LDA using only the parameters with significant intergroup differences, we observed that the difference between controls and HTLV-1 is not too clear than when we used all the 10 posturographic parameters. It reinforces the need to use a bigger number of variables to separate groups with different similarities as we had in the present study.

In the present study, the posturographic measurement was more sensitive to identify subjects with impaired balance control than the BERG balance scale. Similar findings by Prosperini *et al*.^34^ showed that static posturography was more accurate than BBS, a well-established clinical tool for measuring balance in multiple sclerosis and in a different clinical setting. They found that BBS had poor sensitivity, although slightly higher specificity than the COP path when eyes were open, with more than 10% difference in accuracy favoring of static posturography. Some studies done on patients with multiple sclerosis (MS) have also demonstrated balance deficits in minimally impaired MS patients, even in patients with normal clinical balance test, thus suggesting that MS patients may have a subclinical balance disorder unrevealed by conventional balance tests^33^. We suggest the same for HTLV-1, the patients seem to carry no risk of fall according to the BERG balance scale, but on evaluation by force platform, altered parameters were revealed.

By evaluating posture control, we were able to identify functional differences between controls and HTLV-1 infected patients, and thus evaluation of postural control can be considered as an alternative objective and non-invasive tool in the clinical evaluation of these patients.

## Limitations

The sample size could be increased to reach stronger and definitive conclusions. Although the HTLV-1 is not included in the list of neglected diseases, it is not well-investigated when compared to other retroviruses, like HIV. Owing to this, the infected population can be underestimated, and many people might not be aware of being potentially infected.

## Conclusion

Altered posturographic parameters resulting from sensory and neurological impairments were found in the HAM/TSP group when it was compared to HTLV-1 and control group. The damage in postural control was not so evident in HTLV-1 group compared to the control group. Both groups had altered parameters when changed from eyes open to eyes closed. HTLV-1 seems to be an intermediate group between healthy and symptomatic patients. Posturography is an efficient method to quantify and analyze postural control in this group of patients.
